# Blocking the Class I Histone Deacetylase Ameliorates Renal Fibrosis and Inhibits Renal Fibroblast Activation via Modulating TGF-Beta and EGFR Signaling

**DOI:** 10.1371/journal.pone.0054001

**Published:** 2013-01-16

**Authors:** Na Liu, Song He, Li Ma, Murugavel Ponnusamy, Jinhua Tang, Evelyn Tolbert, George Bayliss, Ting C. Zhao, Haidong Yan, Shougang Zhuang

**Affiliations:** 1 Department of Nephrology, Shanghai East Hospital, Tongji University School of Medicine, Shanghai, China; 2 Department of Medicine, Rhode Island Hospital and Alpert Medical School of Brown University, Providence, Rhode Island, United States of America; 3 Department of Laboratory Medicine, Shekou People's Hospital, Shenzhen, Guangdong Providence, China; 4 Department of Surgery, Roger William Medical Center, Boston University Medical School, Providence, Rhode Island, United States of America; INSERM, France

## Abstract

**Background:**

Histone deacetylase (HDAC) inhibitors are promising anti-fibrosis drugs; however, nonselective inhibition of class I and class II HDACs does not allow a detailed elucidation of the individual HDAC functions in renal fibrosis. In this study, we investigated the effect of MS-275, a selective class I HDAC inhibitor, on the development of renal fibrosis in a murine model of unilateral ureteral obstruction (UUO) and activation of cultured renal interstitial fibroblasts.

**Methods/Findings:**

The UUO model was established by ligation of the left ureter and the contralateral kidney was used as a control. At seven days after UUO injury, kidney developed fibrosis as indicated by deposition of collagen fibrils and increased expression of collagen I, fibronectin and alpha-smooth muscle actin (alpha-SMA). Administration of MS-275 inhibited all these fibrotic responses and suppressed UUO-induced production of transforming growth factor-beta1 (TGF-beta), increased expression of TGF-beta receptor I, and phosphorylation of Smad-3. MS-275 was also effective in suppressing phosphorylation and expression of epidermal growth factor receptor (EGFR) and its downstream signaling molecule, signal transducer and activator of transcription-3. Moreover, class I HDAC inhibition reduced the number of renal tubular cells arrested in the G2/M phase of the cell cycle, a cellular event associated with TGF-beta1overproduction. In cultured renal interstitial fibroblasts, MS-275 treatment inhibited TGF-beta induced phosphorylation of Smad-3, differentiation of renal fibroblasts to myofibroblasts and proliferation of myofibroblasts.

**Conclusions and Significance:**

These results demonstrate that class I HDACs are critically involved in renal fibrogenesis and renal fibroblast activation through modulating TGF-beta and EGFR signaling and suggest that blockade of class I HDAC may be a useful treatment for renal fibrosis.

## Introduction

The pathogenesis of chronic kidney disease (CKD) involves a complex interaction of hemodynamic and inflammatory processes that leads to a final common pathway to renal fibrosis, which is characterized by activation of renal fibroblasts and accumulation of excessive amounts of extracellular matrix (ECM) proteins [1], [2]. It has been demonstrated that transforming growth factor-beta1 (TGF-beta1) is the most important growth factor in inducing fibrogenesis. Elevated TGF-beta1 expression has been noted in animal models of renal fibrosis and in patients with glomerulonephritis and diabetic nephropathy [3], [4]. Inhibition of TGF-beta signaling, either pharmacologically or genetically, attenuated tubulointerstitial fibrosis in renal injury models [5], [6]. TGF-beta1 exerts its biological functions through interaction with TGF-beta receptors, which are composed of type I (TbetaRI) and type II (TbetaRII) receptors [7], [8]. TGF-beta1 binding to TbetaRII, results in the recuitment, phosphorylation, and concomitant activation of TbetaRI. Activated TbetaRI induces phosphorylation of Smad3, which forms a complex with Smad4, and then is translocated to nuclei where it drives expression of TGF-beta target genes such as collagens.

Activation of epidermal growth factor receptor (EGFR) has also been reported to be involved in renal fibrogenesis. For example, mice overexpressing a dominant negative EGFR construct exhibited significantly less tubulointerstitial injury in the kidney compared with wild type littermates after subtotal renal ablation or following chronic angiotensin II infusion [9], [10]. A decrease in renal fibrosis was observed in mice with deletion of EGFR in proximal renal tubular cells after angiotensin II infusion or in Waved-2 mice that have reduced EGFR kinase activity after ureteral obstruction [11], [12]. Furthermore, pharmacologic blockade of EGFR with gefitinib or erlotinib inhibits renal deterioration and fibrogensis induced by angiotensin II, unilateral ureteral obstruction (UUO) or 5/6 renal nephrectomy [9], [10] [11], [12].. This suggests that EGFR is critically involved in the development of renal fibrosis.

Histone deacetylases (HDACs) are a family of enzymes that remove acetyl groups from histone and non-histone proteins and play an important role in regulating gene transcription and protein functions [13]–[19]. Based on their homology to yeast HDACs, HDACs are divided into class I (HDAC1, 2, 3 and 8); class II (HDAC4, 5, 6, 7, 9 and 10); class III (SIRT1–7); and class IV (HDAC11) [20]. The first two classes of HDACs are considered “classical” HDACs and are the major targets of current pan-HDAC inhibitors [i.e. Trichostatin A, vorinostat] in therapies against cancers [20], [21]. Besides anticancer activities, these pan-HDAC inhibitors also exhibit inhibitory effects against tissue fibrosis in the heart [22], [23], liver [24], [25], skin [26] and kidney [27], [28], [29], suggesting the potential of HDACs as targets for the treatment of chronic fibrotic diseases. However, recent genetic studies indicated that class I and class II HDACs have opposite effects in cardiac hypertrophy, during which class II HDACs repress whereas class I HDACs promote the hypertrophic response [30], [31]. Furthermore, pan-HDAC inhibitors have been shown to have deleterious effects on the ventricular failure in a model of pulmonary artery banding [32]. Given such advere effects of pan-HDAC inhibitors, Cavasin et al., recently examined the therapeutic effect of MS-275 (Entinostat), a selective class I inhibitor, on cardiopulmonary remodeling in a preclinical model of pulmonary hypertension and demonstrated that adminstration of MS-275 (Entinostat) can potently suppress cardiopulmonary remodeling without damaging cardiac ventricular function [33]. Thus, selective inhibition of class I HDACs may have more favorable effect than general HDAC inhibition in clinical practice.

Recently, we have demonstrated that Trichostatin A, one of pan-HDAC inhibitors is effective in attenuating renal fibrosis and suppressing activation of renal fibroblasts [27], [28], [29]. However, the efficacy of selective class I HDAC inhibitors in renal fibrosis remains unclear. In this study, we assessed the effect of MS-275 on the development of renal fibrosis in a murine model of UUO and on the activation and proliferation of cultured renal interstitial fibroblasts. Moreover, we explored the mechanism by which MS-275 exerts its anti-fibrotic effects, with the focus on the TGF-beta and EGFR signaling.

## Results

### Inhibition of class I HDAC by MS-275 attenuates the development of renal fibrosis after UUO injury

**Figure 1:**
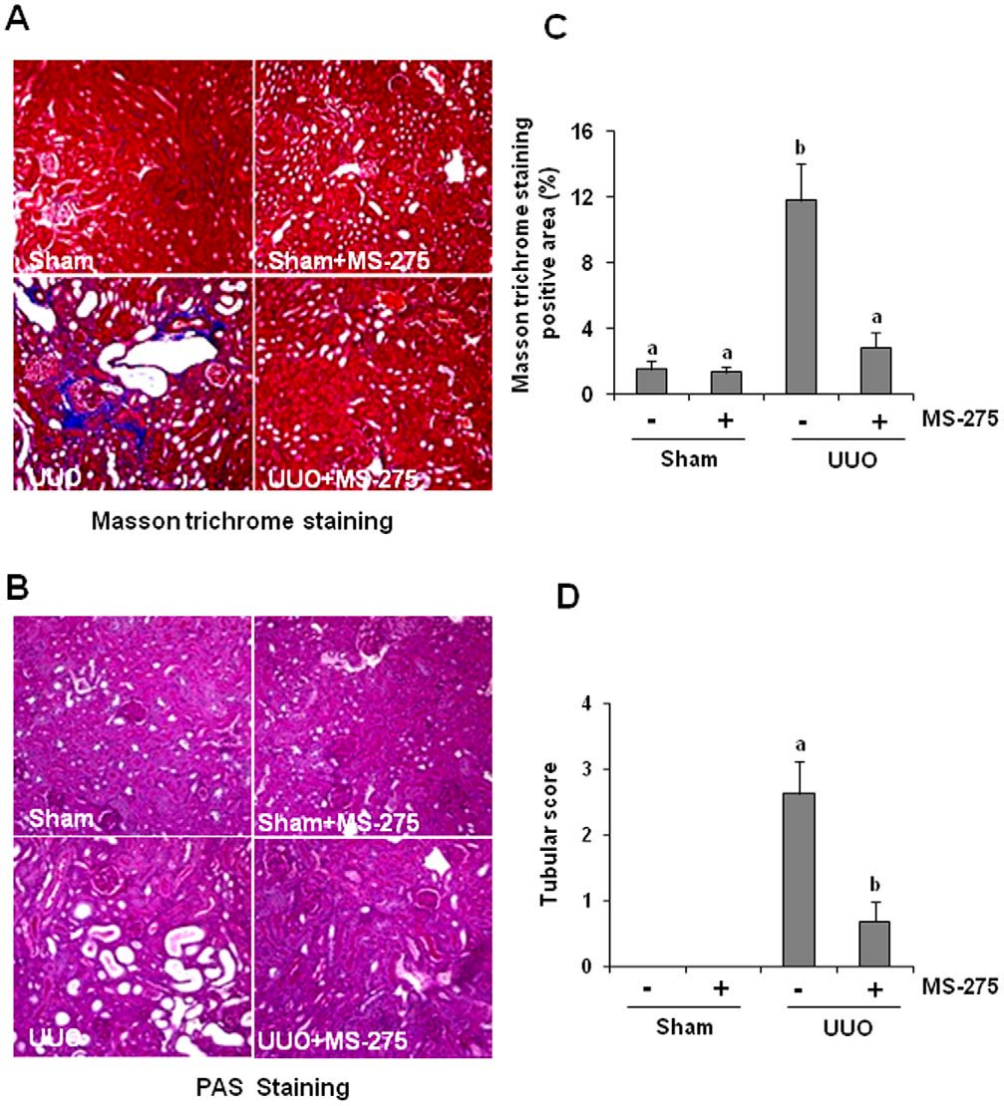
MS-275 reduces renal injury and the deposition of ECM in obstructed kidneys. (A) Photomicrographs illustrating masson trichrome (A) or PAS (B) staining of kidney tissue after various treatments. The Masson trichrome-positive tubulointerstitial area (blue)(C) relative to the whole area from 10 random cortical fieldswas analyzed andthe kidney damage score was also conducted in 10 random fields of each groups (D) (100 X) Data are represented as the mean ± SEM (n = 6). Means with different superscript letters are significantly different from one another (*P*<0.05).

To determine whether class I HDACs mediate renal fibrogenesis, we examined the effect of MS-275 on the development of renal fibrosis a murine model of UUO. As shown in Figure 1, UUO injury induced expression of interstitial collagen fibrils, as indicated by Masson trichrome staining and administration of MS-275 at a dose of 20 mg/kg resulted in a dramatic attenuation of collagen fibril deposition. A semi-quantitative analysis of Masson trichrome-positive areas reveals a 6-fold increase of ECM in obstructive kidneys compared to sham-operated kidneys. MS-275 treatment resulted in approximately 90% less renal fibrosis in the obstructive kidney compared to the kidney injured by UUO alone. In addition, there is also less tubular dilation in the injured kidney after MS-275 treatment as demonstrated by Periodic Acid Schiff (PAS) staining (Figure 1B, 1D). These results illustrate that class I HDACs play a critical role in the regulation of renal fibrosis development and MS-275 is a potent anti-fibrotic agent.

### MS-275 inhibits expression of alpha-SMA, collagen type I and fibronectin in the obstructed kidney

**Figure 2:**
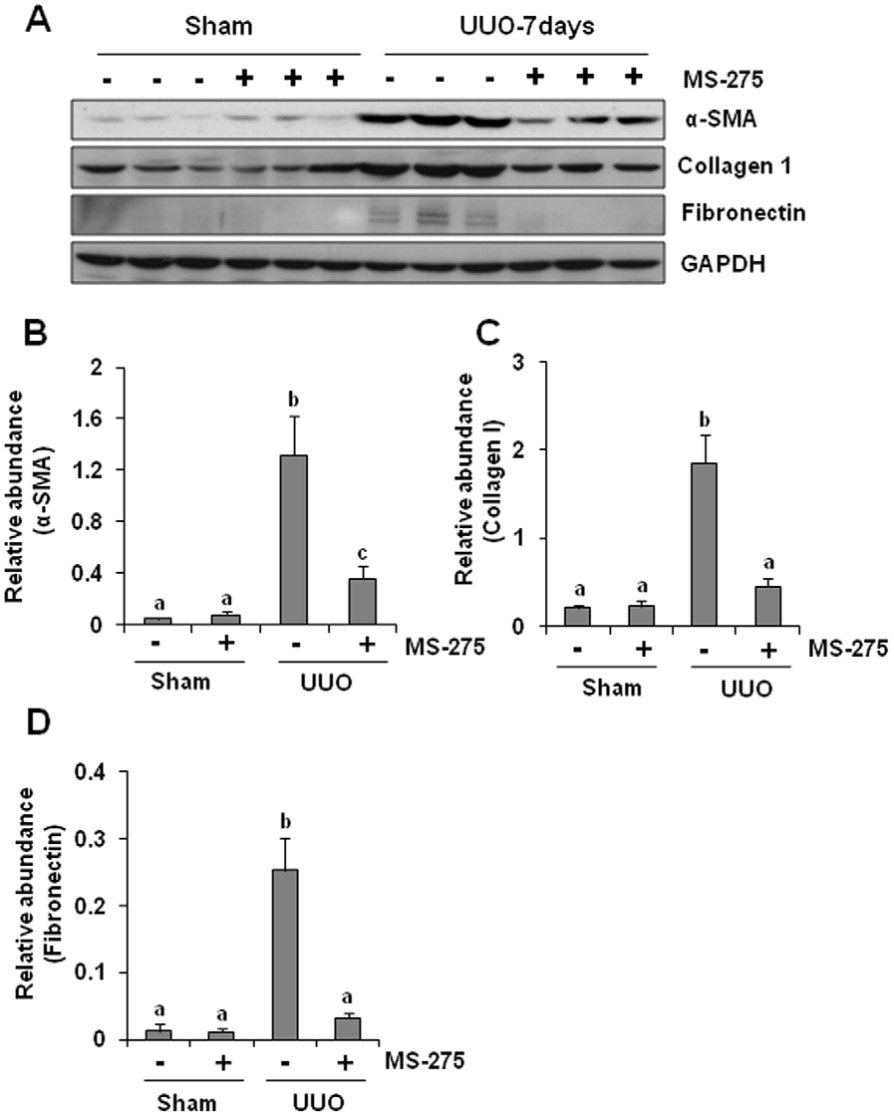
MS-275 inhibits the expression of collagen I, fibronectin, and alpha-SMA in obstructed kidneys. Kidney tissue lysates were subject to immunoblot analysis with specific antibodies against fibronectin, collagen I, alpha-SMA, or GAPDH (A). Expression levels of alpha-SMA (B), collagen I (C) or fibronectin (D), were quantified by densitometry and normalized with GAPDH. Data are represented as the mean ± SEM (n = 6). Means with different superscript letters are significantly different from one another (*P*<0.05).

Activation of renal interstitial fibroblasts and overproduction of ECM proteins are considered pivotal events in the pathogenesis of chronic renal fibrosis [8]. To confirm the anti-fibrotic effect of MS-275, we further examined the effect of MS-275 on the expression of alpha-smooth muscle actin (alpha-SMA), a hallmark of fibroblast activation, and fibronectin and collagen type 1, two major ECM proteins in obstructed kidneys. A basal level of alpha-SMA and abundant collagen type 1 were detected in the kidney tissue of sham-operated mice and their expression was dramatically increased after UUO injury. Treatment with MS-275 largely blocked UUO-induced alpha-SMA expression and decreased collagen type 1 expression to its basal level. This agent however did not affect the basal level of collagen type 1 and alpha-SMA expression in sham-operated kidneys (Figure 2A–C). Although fibronectin was not detected in the kidney tissue of sham-operated kidney, UUO injury resulted in a 25-fold increase in its expression compared with that in the control kidney. Treatment with MS-275 abolished this response (Figure 2A, 2D). Collectively, these data suggests that class I HDACs are critically involved in the activation of renal interstitial fibroblasts and production of ECM proteins in the kidney after UUO injury.

### MS-275 treatment induces expression of acetylated histone H3 in the kidney

**Figure 3:**
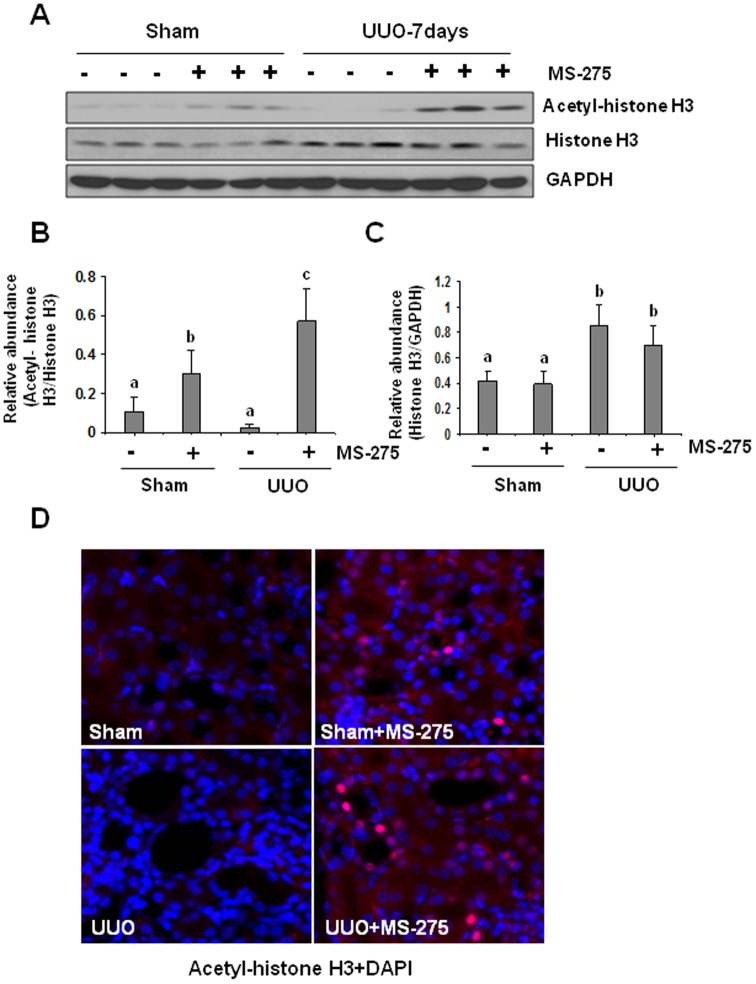
MS-275 enhances expression of acetyl-histone H3 in obstructed kidneys. Kidney tissue lysates were subject to immunoblot analysis with specific antibodies against acetyl-histone H3 (A). Expression levels of acetyl-histone H3 and histone H3 were quantified by densitometry and normalized with histone H3 (B) and GAPDH, respectively (C). Data are represented as the mean ± SEM (n = 6). Means with different superscript letters are significantly different from one another (*P*<0.05). Photomicrographs illustrate acetyl-histone H3 with immunofluorescent staining after various treatments as indicated (D).

Since HDAC activation can cause deacetylation of histone and some non-histone proteins, HDAC inhibition would be expected to induce histone hyperacetylation due to unopposed histone acetyltransferase. To ensure the efficacy of MS-275 in suppressing HDACs, we first examined the expression of acetylated histone H3 by immunoblot analysis. Acetylated histone H3 was barely detectable in sham-operated kidneys, and UUO injury did not significantly increase its expression. MS-275 treatment induced histone H3 hyperacetylation in both sham-operated and UUO injured kidneys with a 5-fold increase of its expression in the obstructed kidney related to that in sham-operated kidneys (Figure 3A, 3B). Total histone H3 level was also slightly increased in the UUO injuried kidney compared with that in non-injured kidneys (Figure 3C). Immunostaining of the kidney tissue indicates that acetylated histone H3 was mainly expressed in the sham-operated and UUO injured kidneys with MS-275 treatment (Figure 3D). These data indicate that 20 mg/kg of MS-275 is sufficient to block class I HDACs, resulting in elevation of protein acetylation levels.

### MS-275 suppresses epithelial cell cycle arrest at G2/M phase and subsequent production of TGF-beta1 in the obstructed kidney

**Figure 4:**
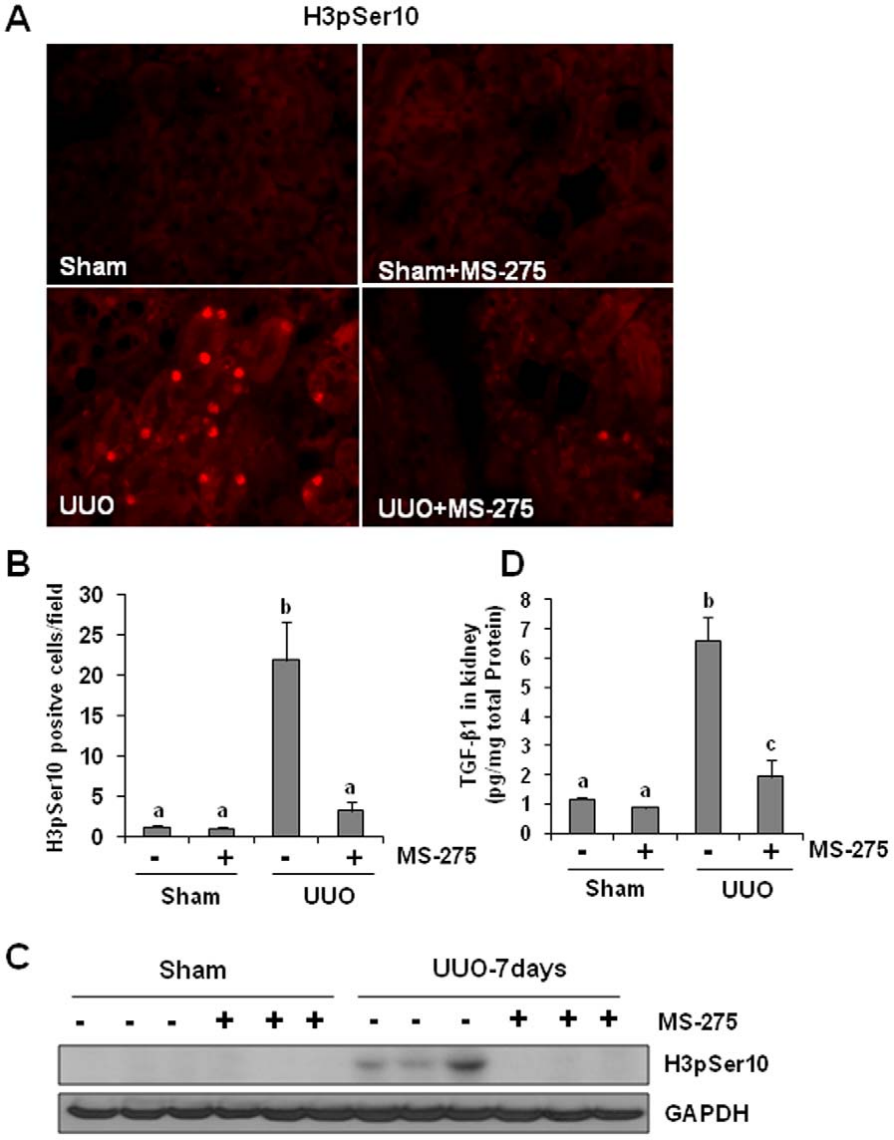
Effect of MS-275 on epithelial cell cycle arrest at G2/M phase and the production of TGF-beta1 in the obstructed kidneys. Kidney tissue was collected on day 7 after ureteral obstruction. Photomicrographs illustrate p-histone H3 at serine 10 (H3pSer10, red color) with immunofluorescent staining after various treatments as indicated (A). H3pSer10-positive cells were accounted from 10 random cortical fields (200 X) (means ± SEM) (B). Tissue lysates were subject to immunoblot analysis with specific antibodies against H3pSer10 (C). Protein from kidney tissues from sham-operated or obstructed kidneys with/without MS-275 administration was subjected to the determination of TGF-beta1 levels by the enzyme-linked immunosorbent assay (ELISA) (D). Data are represented as the means ± SEM (n = 6). Means with different superscript letters are significantly different from one another (*P*<0.05).

It was reported that renal epithelial cell cycle arrested at the G2/M boundary results in a prominent profibrotic phenotype that produces an excessive amount of TGFbeta1 [34]. Given that inhibition of class I HDAC activity by MS-275 increased histone H3 acetylation in renal epithelial cells, we tested whether MS-275 inhibits the arrest of epithelial cells at G2/M phase by examining expression of phosphorylated histone H3 at serine 10 (H3pSer10), a hallmark of cells arrested at G2/M stage [34] Immunofluorescent staining showed that H3pSer10 positive cells were not observed in the sham-operated and MS-275 alone-treated kidney, but they were dramatically increased in the kidney after UUO injury. Administration of MS-275 resulted in a significant reduction of this population of cells (Figure 4A, 4B). Immunoblot analysis confirmed that expression of H3pSer10 was increased in the kidney of mice after UUO injury and abolished by MS-275 treatment (Figure 4C). As tubular cells arrested at G2/M phase increase TGF-beta1 production [34], we further examined the effect of MS-275 on the expression of TGF-beta1 using ELISA assay. Figure 4D shows that UUO injury resulted in an increase in the production of TGF-beta, which was largely suppressed by MS-275 treatment. Thus, we suggest that class I HDAC inhibition can decrease TGF-beta1 production, which may occur through inhibition of tubular cells arrested at G2/M phase in the injured kidney.

### MS-275 inhibits TbetaRI expression and Smad3 activation in the obstructed kidney

**Figure 5:**
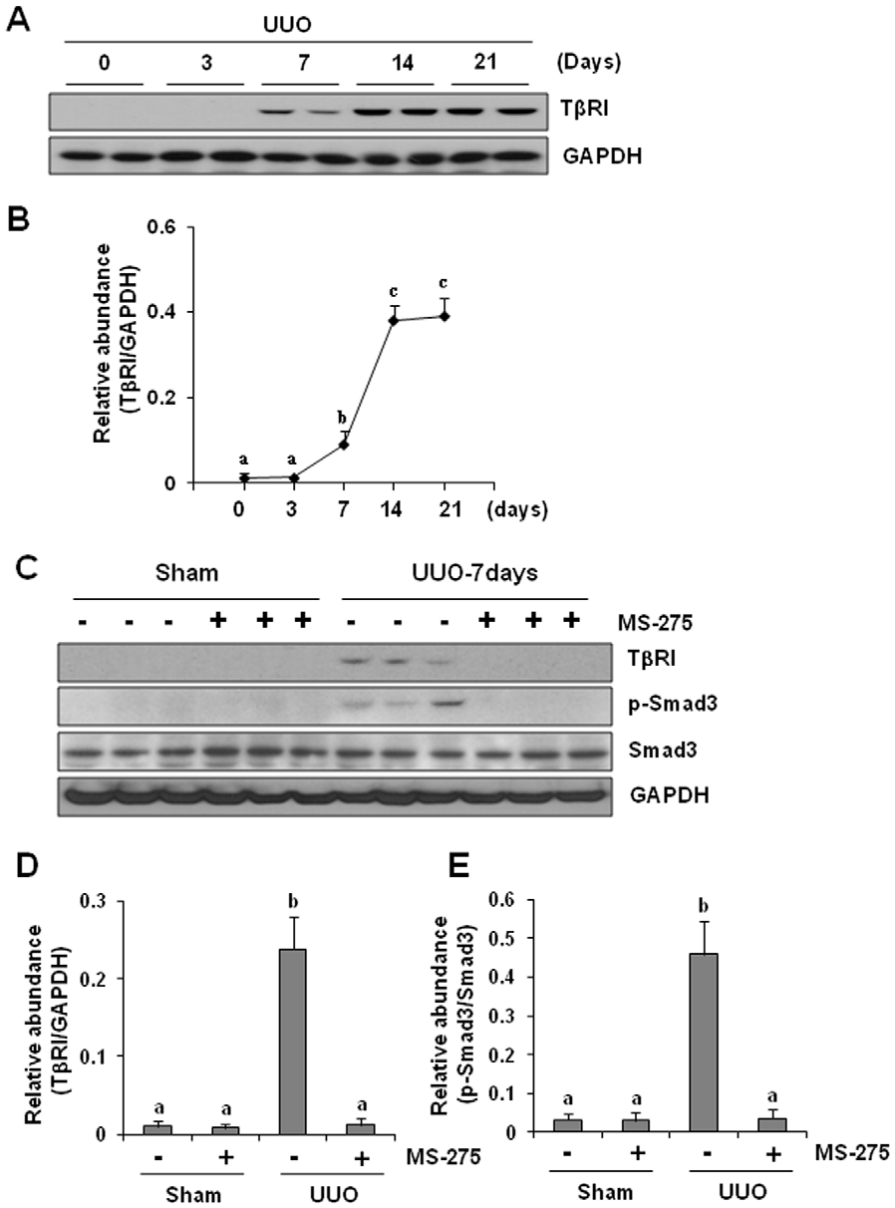
Effect of MS-275 on UUO-induced TbetaRI expression and Smad3 phosphorylation in obstructed kidneys. Kidney tissue was collected at the time points as indicated (A) or on day 7after ureteral obstruction (B), and cell lysates were subject to immunoblot analysis with specific antibodies against TbetaR1 (A, C), p-Smad3 and Smad3, respectively (C). Expression levels of TbetaR1 and p-Smad3 were quantified by densitometry and normalized with GAPDH and Smad3, respectively (D, E). Data are represented as the mean ± SEM (n = 6). Means with different superscript letters are significantly different from one another (*P*<0.05).

An increase in the expression of TGF-beta receptors and activation of the Smad signaling pathway are critical steps for inducing renal fibrogenesis [35], [36]. Among three TGF-beta receptors (TbetaRI, TbetaRII and TbetaRIII), TbetaRI can directly interact with and phosphorylate Smad3, the key mediator of TGF-beta signaling [35], [36]. As such, we examined renal TbetaRI expression and Smad3 phosphorylation after UUO injury. Figure 5A, 5B shows that ureteral obstruction resulted in a time-dependent increase in the expression of TbetaRI, which occurred at 7 days after surgery, reached its maximum level at 14 days and persisted for at least 21 days. Consistently, ureteral obstruction also induced Smad3 phosphorylation. Administration of MS-275 abolished both responses (Figure 5C, 5D, 5E). Although total Smad3 was abundantly observed in sham-operated kidneys, it was not affected by UUO injury or MS-275 treatment (Figure 5C). These data suggest that UUO injury induces a sustained expression of TbetaRI and p-Smad3, and these processes are subjected to regulation by class I HDACs.

### MS-275 inhibits TGF-beta11induced Smad3 phosphorylation in cultured renal interstitial fibroblasts

**Figure 6:**
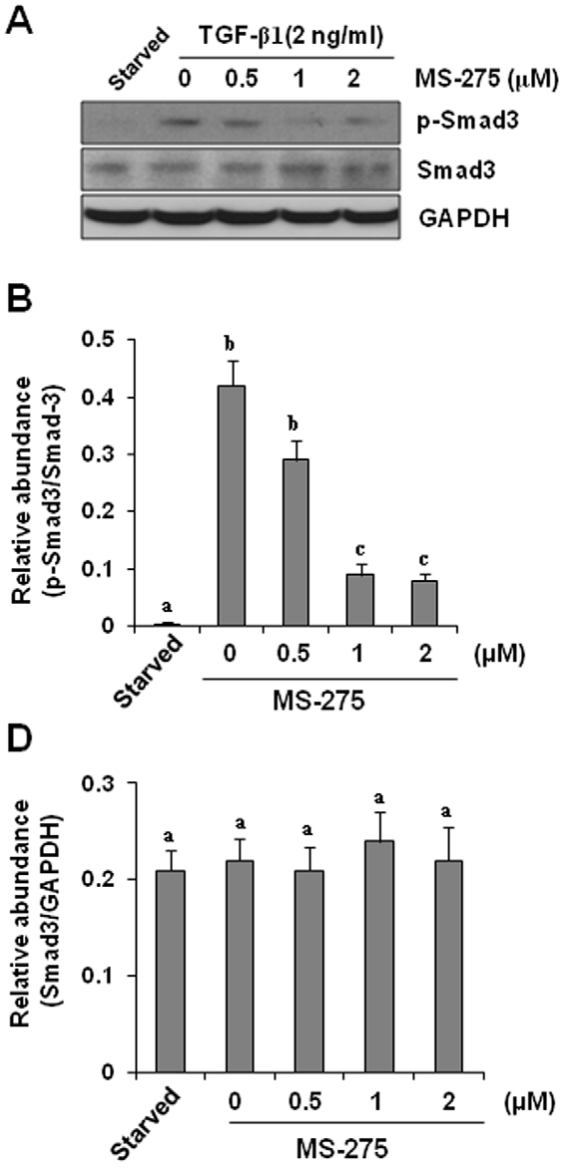
Effect of MS-275 on TGF-beta1-induced phosphorylation of Smad-3 in cultured renal interstitial fibroblasts. Serum-starved NRK-49F cells were incubated with 2 ng/ml TGF-beta1 for 24 h in the presence or absence of MS-275 (0–2 µM). Cell lysates were subject to immunoblot analysis with antibodies to p-Smad3, Smad3, GAPDH (A). Representative immunoblots from three or more experiments are shown. Expression levels of the indicated proteins were quantified by densitometry and normalized with Smad-3 (B) or GAPDH (C). Data are represented as the mean ± SEM. Means with different superscript letters are significantly different from one another (*P*<0.05).

*In vitro* studies have implicated interstitial fibroblasts as the principal mediators of TGF-beta-induced tubulointerstitial fibrosis *in vivo* [36], [37]. To specifically demonstrate the role of class I HDACs in regulation of TGF-beta signaling, we further examined the effect of HDAC inhibition on Smad-3 phosphorylation in NRK-49F, a rat renal interstitial fibroblast line. Exposure of serum-starved NRK-49F to TGF-beta1 resulted in increased phosphorylation of Smad-3. Incubation of NRK-49F with TGF-beta1 in the presence of MS-275 suppressed its phosphorylation whereas expression of total Smad-3 was not affected by this compound (Figure 6A-C), which confirms that class I HDACs inhibition can block TGF-beta signaling in renal interstitial fibroblasts.

### Class I HDACs are required for activation and proliferation of renal interstitial fibroblasts

**Figure 7:**
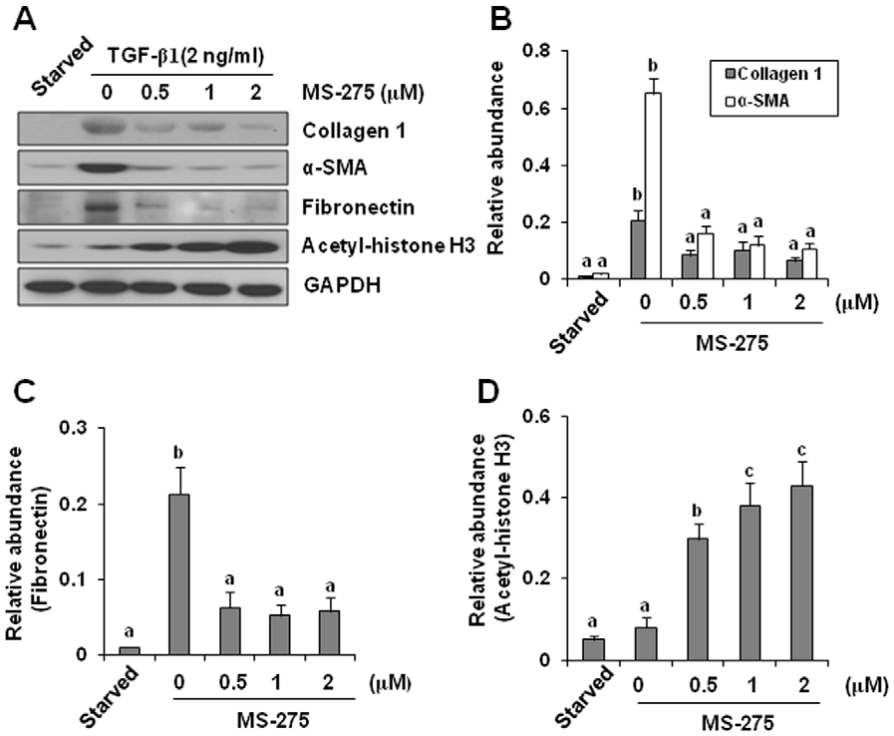
Effect of MS-275 on TGF-beta1-induced expression of collagen 1, alpha-SMA, fibronectin and acetyl-histone H3 in cultured renal interstitial fibroblasts. Serum-starved NRK-49F cells were incubated with 2 ng/ml TGF-beta1 for 24 h in the absence or presence of MS-275 (0–2 µM). Cell lysates were subject to immunoblot analysis with antibodies to the indicated proteins (A). Representative immunoblots from three or more experiments are shown. Expression levels of the indicated proteins were quantified by densitometry and normalized with GAPDH (B, C, D). Data are represented as the mean ± SEM. Means with different superscript letters are significantly different from one another (*P*<0.05).

**Figure 8:**
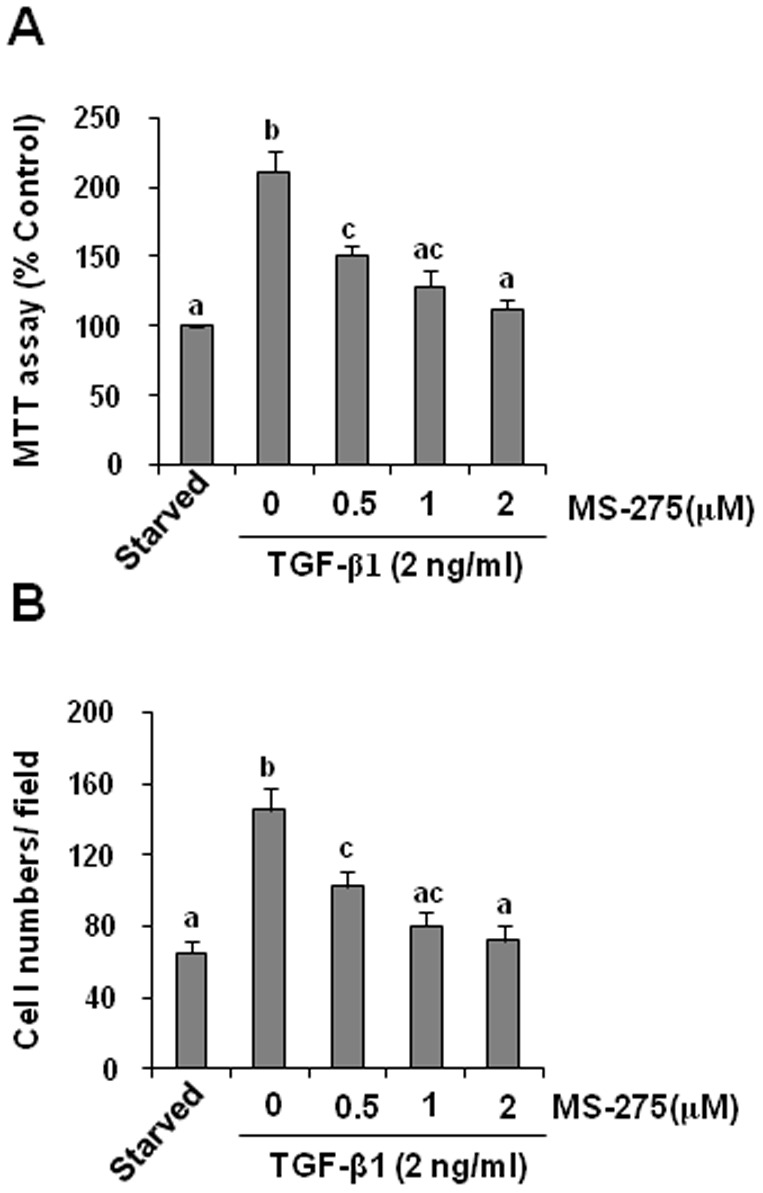
Effect of MS-275 on the proliferation of cultured of renal interstitial fibroblasts. NRK-49F cells were serum-starved for 24 h and then incubated in the presence of TGF-beta1 (2 ng/ml) for 48 h in the absence or presence of 0–2 µM MS-275 (A, B). Cell proliferation was assessed by the MTT assay or counting cells (B). Data are represented as the mean ± SEM. Means with different superscript letters are significantly different from one another (*P*<0.05).

It has been reported that TGF-beta1 can induce differentiation of renal fibroblasts into myofibroblasts which is characterized by expression of collagen1, fibronectin, and alpha-SMA [36] and cell proliferation, we thus further examined the effect of MS-275 on activation and proliferation of renal interstitial fibroblasts in vitro. Exposure of serum-starved NRK-49F cells to TGF-beta1 resulted in increased expression of collagen 1, fibronectin, and alpha-SMA. Treatment with MS-275, suppressed all these events in a dose dependent manner with the maximum inhibition at 2 µM (Figure 7A–C). Effective inhibition of MS-275 on HDACs was demonstrated by a dose-dependent up-regulation of acetyl-histone H3 (Figure 7A, 7D). In addition, MS-275 was also able to block proliferation of NRK-49F as demonstrated by decreased cell numbers (Figure 8A, 8B). These data, together with the inhibitory effects of MS-275 on the expression of alpha-SMA and extracellular matrix proteins in the injured kidney, support the critical role of class I HDACs in mediating activation and proliferation of renal interstitial fibroblasts.

### MS-275 inhibits phosphorylation and expression of EGFR expression in the obstructed kidney

**Figure 9:**
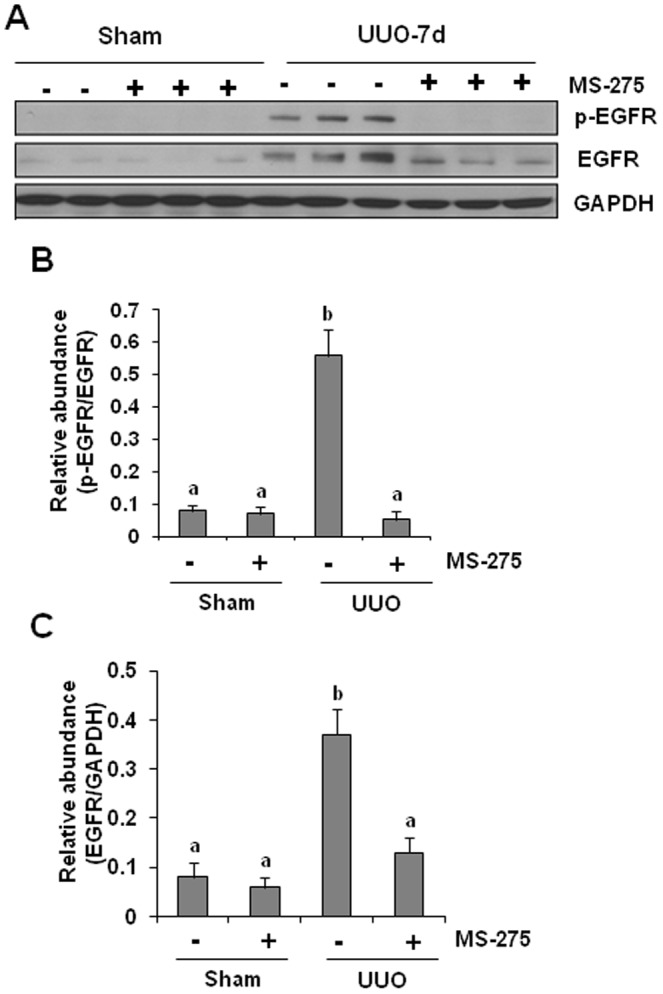
Effect of MS-275 on UUO-induced expression and phosphorylation of EGFR in obstructed kidneys. Kidney tissue lysates were subject to immunoblot analysis with specific antibodies against p-EGFR, or EGFR (A). Expression levels of p-EGFR (B), or EGFR (C) were quantified by densitometry and normalized with EGFR or GAPDH, respectively. Data are represented as the mean ± SEM (n = 6). Means with different superscript letters are significantly different from one another (*P*<0.05).

Previous studies have shown that EGFR activation contributes to the development of renal fibrosis following a prolonged ischemic injury, renal ablation, and chronic agiotensin II infusion [9], [10]. Our recent studies also demonstrated that UUO injury induced a persistent expression and phosphorylation of EGFR, which was critically implicated in renal fibrogenesis [38]. Here we examined whether MS-275 would affect EGFR activation and expression in this model. As shown in Figure 9A, 9B, 9C, MS-275 administration abolished EGFR phosphorylation and also significantly reduced EGFR expression in the obstructive kidney. Therefore, MS-275 is also effective in suppressing EGFR signaling.

### MS-275 inhibits phosphorylation of STAT3 in the kidney following obstruction

**Figure 10:**
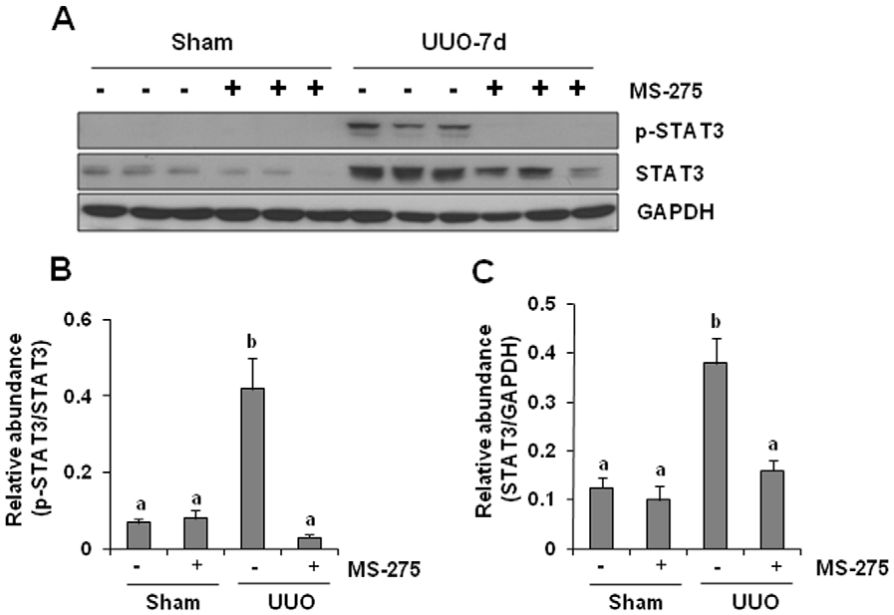
Effect of MS-275 on phosphorylation of STAT3 in the obstructed kidneys. Kidney tissue lysates were subject to immunoblot analysis with specific antibodies against phospho-STAT3 (p-STAT3), or STAT3(A). Expression levels of p-STAT3, or STAT3 were quantified by densitometry. Activated STAT3 (B) was depicted with p-STAT3/STAT3 ratios. Protein levels of STAT3 (C) was normalized with GAPDH. Data are represented as the mean ± SEM (n = 6). Means with different superscript letters are significantly different from one another (*P*<0.05).

STAT3 (signal transducer and activator of transcription 3) signaling pathway is activated in the obstructed kidney and associated with development of renal fibrosis or production of profibrotic cytokines such as TGF-beta1 [39], [40]. We examined the effect of MS-275 on the phosphorylation of STAT3 in the obstructed kidneys. UUO injury induced STAT3 phosphorylation and also increased total STAT3 levels. Administration of MS-275 completely blocked STAT3 phosphorylation and largely suppressed its expression (Figure 10A–C). These data suggest that HDACs also play roles in regulating phosphorylation and expression of STAT3.

### Effect of MS-275 on leukocyte infiltration in the obstructive kidney

**Figure 11:**
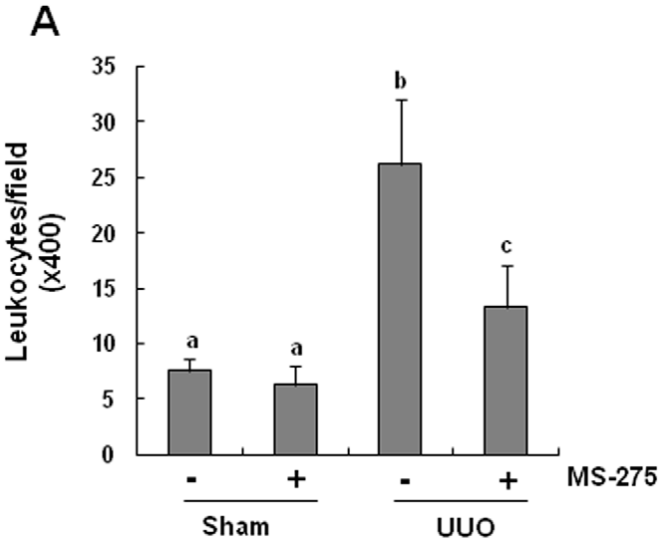
Effect of MS-275 on accumulation of leukocytes in the obstructed kidney. Kidney tissue was collected on day 7 after ureteral obstruction, and naphthol-AS-D-chloroacetate esterase staining was performed and leukocytes were counted in three random fields of each sample, and 10 fields (×200) were analyzed for each condition. Data are represented as the mean ± SEM (n = 6). Means with different superscript letters are significantly different from one another (*P*<0.05).

It is well known that accumulation of inflammatory cells contribute to the development of renal fibrosis in human and animal models of CKD [41]. We therefore also examined the effect of MS-275 on leukocyte infiltration. Staining of kidney sections with naphthol-AS-D-chloroacetate esterase showed prominent interstitial infiltration of neutrophils and monocytes after obstructive injury. MS-275 treatment reduced leukocyte infiltration to basal levels (Figure 11). These data suggest that activation of class I HDACs contributes to leukocyte infiltration in UUO-induced renal fibrosis.

### UUO injury alters the expression of class I HDACs

It has been reported that both mRNA and protein levels of HDACs1and 2 are increased, while HDAC3 mRNA is unaltered in the obstructed kidney [42]. To further understand the expression file of class I HDACs in the UUO injured kidney, we examined expression of all the class I HDAC isoforms at their protein levels. Our results indicated that all the four isoforms of class I HDAC (1, 2, 3, 8) are expressed in the control kidney and that UUO injury resulted in increased expression of HDAC1 and HDAC2, decreased HDAC3, and unchanged HDAC8 in the kidney (Figure S1). These results indicate different expression of class I HDAC isoforms in the obstructed kidney and suggest the important implication of HDAC1 and HDAC2 in mediating renal fibrogenesis after chronic kidney injury.

## Discussion

In this study, we demonstrated that blocking class I HDACs with MS-275 attenuates development of renal fibrosis and inhibits activation and proliferation of renal interstitial fibroblasts. Further, MS-275 treatment effectively inhibits TGF-beta1 production, TbetaRI and EGFR expression as well as Smad3 and STAT3 phosphorylation. These results extend our previous observations [27], [28], [29] by providing the first evidence that inhibition of class I HDACs protects the kidney against fibrogenesis and TGF-beta and EGFR signalings are the targets of HDAC inhibitors.

Given the central role of TGF-beta signaling in the development of renal fibrosis, inhibition of TGF-beta signaling might serve as a novel mechanism by which HDAC inhibitors attenuate renal fibrogenesis. In this pathway, TGF-beta1 binding to TbetaRII results in the formation of a receptor complex with TbetaRI, leading to TbetaRI phosphorylation. Subsequently, TbetaRI propagates profibrotic signal via phosphorylation/activation of Smad-3 [36]. Inhibition of TGF-beta signaling at distinct levels including TGF-beta production, TbetaRI expression, and Smad-3 phosphorylation in the obstructed kidney suggests a critical role of class I HDACs in regulating this signaling pathway. How HDAC inhibition interferes with these components in TGF-beta signaling pathway is currently unclear. A recent study showed that TGF-beta1 is mainly produced in renal epithelial cells arrested in the G2/M phase of the cell cycle, and this population of cells is increased after injury [34]; thus it is possible that class I HDAC inhibition may reduce TGF-beta1 production through inhibition of tubular cells arrested in the G2/M phase of the cell cycle. We tested this hypothesis by immunostaining and Western blot analysis of expression of phospho-histone at serine 10, a hallmark of cells arrested at G2/M stage [34] in the absence or presence of MS-275. Our results show that MS-275 treatment decreased number of cells arrested at G2/M in the obstructed kidney, suggesting that class I HDACs are involved in this process and subsequent production of TGF-beta1. However, we can not rule out the possibility that MS-275 might also suppress TGF-beta1 production by acing on other cell types. In this context, it has been reported that microphages infiltrating the injured kidney also have an ability to produce TGF-beta1 [37] and HDAC inhibition with KBH-A42 can inhibit cytokine production in cultured RAW 264.7 macrophage cells [43]. We also observed that MS-275 treatment inhibits microphage infiltration of the UUO injured kidney, suggesting that MS-275 might be able to regulate production of profibrotic factors through other cell types as well.

Our data indicated that UUO injury induces a persistent increase of TbetaRI, which was abolished by MS-275 treatment, suggesting that class I HDAC inhibition may also suppress the TGF-beta signaling through TbetaRI down-regulation. The mechanism by which class I HDAC inhibition suppresses TbetaRI expression is not understood. As most of growth factor receptors, including TGF-beta1 receptor, can be down-regulated by an ubiquitin-dependent degradation [44], [45], [46] it is possible that inhibition of HDACs interferes with TbetaRI expression by promoting the process of ubiquitination. In this regard, we showed that MS-275 administration was also able to suppress expression/phosphorylation of EGFR in the obstructive kidney. In addition, it was reported that vorinostat, a pan-HDAC inhibitor, induces EGFR ubiquitination in tumor cell lines by predominantly targeting lysosome-degradation [47]. Genetic inhibition of HDAC6 also accelerates segregation of EGFR from early endosomes to the late endosomal and lysosomal compartments for degradation [48], [49]. However, HDAC6 is an isoform of the class II HDACs, which is not sensitive to MS-275. As such, it is assumed that one or more class I HDAC isozymes may be involved in the regulation of endocytic trafficking and degradation of TbetaRI and EGFR. Additional experiments are needed to elucidate the molecular basis by which HDAC inhibition induces TbetaRI and EGFR degradation and identify the class I HDAC isoform(s) responsible for this event.

Phospho-Smad-3 is highly expressed in the obstructed kidney, and is necessary for transducing TGF-beta receptor activation to renal fibrogenesis. A complete blockage of Smad-3 phosphorylation by MS-275 in the injured kidney suggests that class I HDACs play a critical role in the regulation of this signaling pathway. In line with this observation, we demonstrated that MS-275 dose-dependently inhibited Smad-3 phosphorylation in cultured renal interstitial fibroblasts, which is concomitant with decrease of expression of ECM proteins and alpha-SMA. As our recent studies have shown that phospho-Smad-3 is primarily located in renal myofibroblasts in the fibrotic kidney [38], we suggest that class I HDAC inhibition is able to desensitize the response of renal fibroblasts to TGF-beta1 stimulation. Given that HDAC inhibition reduced TGF-beta1 production and suppressed TbetaRI expression, it is likely that Smad-3 dephosphorylation is secondary to inhibition of those upstream events of Smad-3 in the TGF-beta1 signaling.

As activation of STAT3 signaling pathway is the consequence of activation of multiple growth factor and cytokine receptors, and our data show that MS-275 treatment decreased expression and/or phosphorylation of TbetaRI and EGFR, we further examined the effect of MS-275 on UUO-induced STAT3 phosphorylation. Like the effect of MS-275 on Smad-3, this inhibitor also suppresses STAT3 phosphorylation in the obstructed kidney. Additionally, MS-275 treatment reduces STAT3 protein expression. This suggests that class I inhibition inactivates STAT3 through interfering with both STAT3 expression and phosphorylation. However, we can not exclude the possibility that MS-275-mediated STAT3 dephosphorylation is not acting on itself, but through suppression or downregulation of its upstream activators including TbetaRI and EGFR. Our future studies will address these issues.

HDAC inhibitors have been primarily developed for hematological and oncological indications [21], [50]. They have been shown to induce growth arrest, differentiation, and apoptosis in malignant cells [21]. In addition to their anticancer actions, clinical and preclinical studies have revealed that HDAC inhibitors potently suppress inflammation, fibrosis, and restenosis. However, most of these results were obtained by using pan-HDAC inhibitors, which inhibit both class I/II HDACs. MS-275 is a new generation class I HDAC inhibitor and preferentially inhibits HDAC1 and HDAC3 and no inhibitory activity towards HDAC8 [50], [51]. Clinical studies of MS-275 in tumors have shown many promising properties in addition to its anti-tumor effects, including good tolerance, good oral bioavailability and linear pharmacokinetics [14], [52]. Our current studies showed that MS-275 is also effective in inhibiting renal fibrogenesis and renal fibroblast activation after UUO injury, suggesting its clinical application as an anti-fibrotic agent.

In summary, we demonstrate for the first time that inhibition of class I HDAC by MS-275 attenuates renal fibrosis and suppresses activation of renal interstitial fibroblasts. The antifibrotic effect of HDAC inhibition is associated with down-regulation of TGF-beta and EGFR signalings. Therefore, MS-275 and other class I HDAC inhibitors may hold therapeutic potential for fibrotic kidney diseases.

## Materials and Methods

### Chemicals and antibodies

Antibodies to p-Smad3, Smad3, H3pSer10, Histone H3, p-EGFR, p-STAT3, STAT3 and Acetyl-Histone H3 were purchased from Cell Signaling Technology (Danvers, MA). Antibodies to EGFR, HDAC1, HDAC2, HDAC3, HDAC8, fibronectin, collagen 1(A2), GAPDH and TbetaRI were purchased from Santa Cruz (Santa Cruz, CA). MS-275, antibody to alpha-SMA, the naphthol-AS-D-chloroacetate esterase kit, and all other chemicals were purchased from Sigma (St. Louis, MO). TGF-beta1 enzyme-linked immunosorbent assay (ELISA) kit was from R&D systems (Minneapolis, MN).

### Cell culture and treatments

Rat renal interstitial fibroblasts (NRK-49F) were cultured in Dulbecco's modified eagle's medium (DMEM) (Sigma-Aldrich, St. Louis, MO) containing 5% fetal bovine serum (FBS), 0.5% penicillin and streptomycin in an atmosphere of 5% CO_2_ and 95% air at 37°C. To determine the effects of MS-275 on fibroblast activation, MS-275 was directly added to sub-confluent NRK-49F cells and then incubated for the indicated time as described in Figure legends. For TGF-beta1 treatment, NRK-49F cells were starved for 24 h by incubation with 0.5% FBS containing DMEM and then exposed to TGF-beta1 (2 ng/ml) for 24 h.

### Unilateral ureteral obstruction (UUO) models and MS-275 treatment

The UUO model was established in male C57 black mice that weighed 20–25 g (Jackson Laboratory, Bar Harbor, ME) as described in our previous study [27]. Briefly, the abdominal cavity was exposed via a midline incision and the left ureter was isolated and ligated. The contralateral kidney was used as a control. To examine the effects of MS-275 on renal fibrosis after UUO injury, MS-275 (20 mg/kg) in 50 µl of DMSO was immediately administered, i.p after ureteral ligation and then given every other day at the same dose. Selection of this dose of MS-275 was according to the previous report [53]. For the UUO alone group, mice were injected with an equivalent amount of DMSO. Six mice were used in each group [39]. The animals were sacrificed by cervical dislocation under adequate anesthesia (inaction, 70mg/kg, IP) and the kidneys were removed at day 7 for protein analysis and histological examination.

### Immunofluorescent and histochemical staining

Immunofluorescent and immunohistochemical staining were performed according to the procedure described in our previous studies [27]. Renal tissue was fixed in 4.5% buffered formalin, dehydrated, and embedded in paraffin. For general histology, sections were stained with PAS. For immunofluorescent staining, primary antibodies against acetyl-Histone H3 (1∶100), p-Histone H3(Ser10) (1∶200), and fluorescent-conjugated secondary antibodies (1∶500) were applied to the sections. For assessment of renal fibrosis, Masson trichrome staining was performed according to the protocol provided by the manufacture (Sigma, St. Louis, MO). The collagen tissue area (blue color) was quantitatively measured using Image Pro-Plus software (Media-Cybernetics, Silver Spring, MD, USA) by drawing a line around the perimeter of positive staining area, and the average ratio to each microscopic field (200×) was calculated and graphed. For detection of macrophages, naphthol-AS-D-chloroacetate esterase staining was performed according to our previous protocol [39]. The positive stained leukocytes were counted in each field (200×) and the average number from 10 fields was graphed.

### Immunoblot analysis

Immunoblot analysis of NRK-49F cells and tissue samples were conducted as described previously [27]. The densitometry analysis of immunoblot results was conducted with NIH Image software (National Institutes of Health, Bethesda, MD).

### Measurement of TGF-beta1 levels

To measure renal TGF-beta1 levels, kidneys from mice were homogenized in the extraction buffer containing 20 mM Tris–HCl, pH 7.5, 2 M NaCl, 0.1% Tween 80, 1 mM ethylenediamine tetraacetate, and 1 mM phenylmethylsulfonyl fluoride, and the supernatant was recovered after centrifugation at 19000 g for 20 min at 4°C. Renal tissue TGF-beta1 level was determined by using the commercial Quantikine TGF-beta1 ELISA kit in accordance with the protocol specified by the manufacturer (TGF-beta1 ELISA kit, R&D systems, Minneapolis, MN). Total protein levels were determined by using a bicinconinic acid protein assay kit. The concentration of TGF-beta1 in kidneys was expressed as picograms per milligram of total proteins.

### Statistical analysis

All the experiments were conducted at least three times. Data depicted in graphs represent the means ± SEM for each group. Inter-group comparisons were made using one-way analysis of variance (ANOVA). Multiple means were compared using Tukey's test. The differences between two groups were determined by Student t-test. Statistical significant difference between mean values was marked in each graph. P<0.05 is considered significant.

### Ethics statement

All experimental procedures were performed according to the US Guidelines to the Care and Use of Laboratory Animals and approved by the Lifespan Animal Welfare Committee (Institutional Animal Care and Use Committee) on 09/02/2010 with approval ID 0127-10.

## Supporting Information

Figure S1 **Effect of MS-275 on expression of class I HDAC isoforms in the obstructed kidneys.** Kidney tissue lysates were subject to immunoblot analysis with specific antibodies against HDAC1, HDAC2, HDAC3, HDAC8 or GAPDH (A). Expression levels of individual HDACs were quantified by densitometry and normalized with GADPH (B). Data are represented as the mean ± SEM (n = 6). Means with different superscript letters are significantly different from one another (*P*<0.05).
(TIF)
