# Supplementary figures and images for: Blocking the Class I Histone Deacetylase Ameliorates Renal Fibrosis and Inhibits Renal Fibroblast Activation via Modulating TGF-Beta and EGFR Signaling
Source: PLoS One. 2013 Jan 16;8(1):e54001. doi: 10.1371/journal.pone.0054001 (PMC3546966; doi:10.1371/journal.pone.0054001)

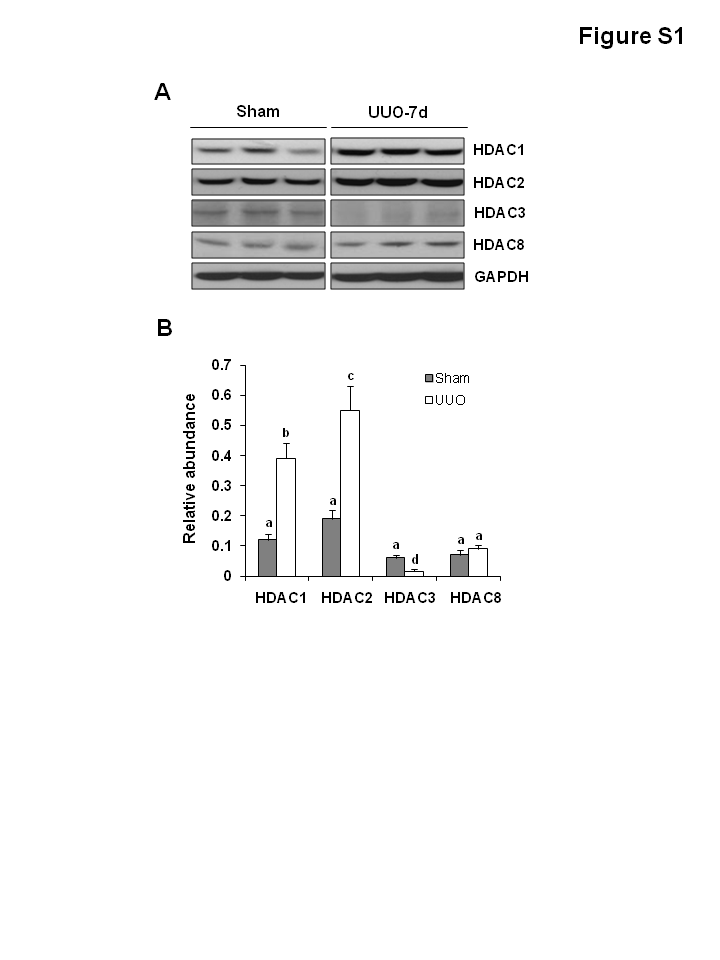

Supplement: Figure S1 — Effect of MS-275 on expression of class I HDAC isoforms in the obstructed kidneys. Kidney tissue lysates were subject to immunoblot analysis with specific antibodies against HDAC1, HDAC2, HDAC3, HDAC8 or GAPDH (A). Expression levels of individual HDACs were quantified by densitometry and normalized with GADPH (B). Data are represented as the mean ± SEM (n = 6). Means with different superscript letters are significantly different from one another (P<0.05). (TIF) [file pone.0054001.s001.tif]
